# Systemic Congestion as a Determinant of Efficacy in Adaptive Servo-Ventilation Therapy: A Retrospective Observational Study

**DOI:** 10.3390/jcm13030674

**Published:** 2024-01-24

**Authors:** Yu Nomoto, Teruhiko Imamura, Koichiro Kinugawa

**Affiliations:** Second Department of Internal Medicine, Toyama University, 2630 Sugitani, Toyama 930-0194, Japan

**Keywords:** non-invasive positive pressure support, hemodynamics, heart failure

## Abstract

Background: The optimal criteria for patient selection in the context of adaptive servo-ventilation (ASV) therapy remain a subject of ongoing investigation. We postulate that baseline plasma volume, assessable through several straightforward clinical parameters, might be correlated with a more pronounced reduction in plasma B-type natriuretic peptide (BNP) levels following mid-term ASV therapy. Methods: We included patients diagnosed with congestive heart failure who had received continuous ASV therapy for a minimum of three months. The primary outcome of interest was the extent of decline in logarithmically transformed plasma BNP levels, defined as a decrease of more than 0.10 during the 3-month ASV treatment period. Results: A total of 66 patients were included in the study. The median age of the cohort was 66 years, with 53 patients (80%) being male. The median plasma volume status at baseline was −16.9%, and patients were categorized into two groups based on this median value. Patients with elevated baseline plasma volume status experienced a statistically significant reduction in plasma BNP levels (*p* = 0.016), whereas those with lower plasma volume exhibited no significant change in BNP levels (*p* = 0.23). A higher baseline plasma volume status was independently associated with a significant reduction in plasma BNP levels, with an adjusted odds ratio of 1.036 (95% confidence interval: 1.01–1.07, *p* = 0.032). Conclusions: The presence of systemic congestion at baseline, quantified by the estimated plasma volume status, may serve as a crucial determinant of the efficacy of ASV therapy, leading to improvements in plasma BNP levels among patients suffering from congestive heart failure.

## 1. Background

The prognosis of patients with chronic heart failure has witnessed substantial improvement, owing to the introduction of novel pharmacological therapies, including the remarkable “Fantastic Four” [1,2,3]. Nevertheless, the persistent challenge of congestion refractory to these pharmacological interventions remains a vexing issue, significantly diminishing patients’ quality of life and exacerbating their overall prognosis [4].

Adaptive servo-ventilation (ASV), as exemplified by the AutoSet-CS device from ResMed, Sydney, Australia, represents a non-pharmacological, non-invasive positive pressure ventilation strategy [5]. This innovative approach serves to stabilize the respiratory system, attenuate the inappropriately activated sympathetic nervous system, and mitigate pulmonary and systemic congestion by modulating preload and afterload [6]. ASV offers promise in carefully selected patients with congestive heart failure, independent of the presence of sleep-disordered breathing [7].

An accumulating body of research has supported the clinical relevance of ASV therapy, highlighting its potential to enhance both mortality and morbidity outcomes in individuals with chronic congestive heart failure [7,8,9,10,11,12,13]. However, a significant setback came with the results of the large-scale, multi-center randomized controlled trial SERVE-HF, which failed to demonstrate a clinical advantage of ASV therapy compared to a placebo [14].

Given these divergent findings, the critical determinant of successful ASV therapy is believed to be optimal patient selection. A series of studies have explored the acute effects of ASV, revealing that the presence of residual congestion was associated with an increase in cardiac output following just a few minutes of ASV support [15,16,17]. This outcome underscores the centrality of addressing congestion in ASV therapy. ASV therapy in patients without congestion seems rather to be harmful and deteriorates hemodynamics by inappropriately reducing preload and declining cardiac output. Yet, the non-invasive assessment of congestion severity, for example, via physical examination, can pose challenges necessitating expert technique. Moreover, the impact of baseline congestion during long-term ASV therapy remains a matter of uncertainty.

Recent advancements have introduced a novel, non-invasive technique for estimating plasma volume (PV) using hematocrit (Ht) and body weight (BW) that is now commercially available [18]. PV reflects the quantity of extravascular fluid and holds a strong association with systemic congestion. Validation through scintigraphy tests and clinical studies has substantiated its ability to accurately gauge systemic congestion [19,20,21,22].

In this context, we posit that patients exhibiting significant systemic congestion (i.e., elevated PV) may derive greater benefit from ASV therapy, leading to a substantial reduction in plasma B-type natriuretic peptide (BNP) levels compared with individuals with little systemic congestion (i.e., lower PV). Our study aimed to evaluate the impact of higher baseline PV levels on the reduction in plasma BNP levels during mid-term ASV therapy, transcending the scope of short-term interventions.

## 2. Methods

### 2.1. Patient Selection

Patients suffering from congestive heart failure who underwent ASV therapy at a prominent academic institution between 2008 and 2022 were considered eligible for this retrospective investigation. Specifically, individuals who received ASV therapy continuously for a duration exceeding three months constituted the study cohort. The decision to employ ASV therapy rested with the attending board-certified cardiologists, guided by their clinical judgment. To elucidate, ASV therapy was prescribed when clinical signs of congestion, persisting despite conventional medical interventions such as diuretics, were evident. The determination of such congestion was established through comprehensive assessment, including physical examination, chest X-ray, and transthoracic echocardiography. All patients provided written informed consent, and the study protocol received approval from the local ethics committee (Ethics Committee, University of Toyama, R2015154, 11 April 2016).

### 2.2. Definition of Heart Failure

Heart failure diagnoses were established following the Framingham criteria by board-certified attending cardiologists [23]. A confirmed diagnosis necessitated the simultaneous presence of at least two major criteria or one major criterion in conjunction with two minor criteria.

Major criteria: paroxysmal nocturnal dyspnea, neck vein distention, rales, radiographic cardiomegaly, acute pulmonary edema, S3 gallop, increased central venous pressure, hepato-jugular reflux, weight loss > 4.5 kg in 5 days in response to treatment.

Minor criteria: bilateral ankle edema, nocturnal cough, dyspnea on ordinary exertion, hepatomegaly, pleural effusion, decrease in vital capacity by one third from maximum recorded, tachycardia.

### 2.3. Study Protocol

Patients with congestive heart failure who sustained ASV therapy for a duration exceeding three months were considered for inclusion in this study. The baseline PV, estimated as detailed below, was designated as the independent variable. Patients were categorized into two groups based on their median value of PV values: a high PV group and a low PV group. These patients were subsequently monitored over three months while receiving ASV support. During the observation period, the values of estimated PV status were blinded to the attending clinicians.

The primary endpoint of interest was characterized as a noteworthy reduction in plasma BNP levels following the three-month ASV therapy. This reduction was defined as a decrease in the logarithm of plasma BNP exceeding 0.10. To illustrate, if the plasma BNP level decreased from 900 pg/mL to 700 pg/mL, the decrease in the logarithm of plasma BNP would be calculated as 0.12, thus qualifying as a substantial reduction. Additionally, changes in various clinical parameters, such as the estimated glomerular filtration rate (eGFR), were examined as secondary outcomes.

### 2.4. PV Estimation

Data regarding BW, body height, and Ht measurements taken immediately before the initiation of ASV therapy were collected from all participants. PV was estimated retrospectively by the expert, who was blinded to the clinical data (Y.N.), utilizing the Hakim Formula, expressed as [18]:

PV (liters) = [1 − Ht (%)] × [a × (b × (lean body mass))].

Here, “a” stands at 1530 for males and 864 for females, whereas “b” is 41.0 for males and 47.2 for females. The lean body mass was computed as follows:

For males: 0.33 × BW (kilograms) + 0.34 × [body height (centimeters)] − 29.5

For females: 0.30 × BW (kilograms) + 0.42 × [body height (centimeters)] − 43.3

The PV status was determined through the following formula:

PV status (%) = [((estimated PV) − (ideal PV))/(ideal PV)] × 100

The ideal PV (liters) was calculated as “a” times BW (kilograms), with “a” being 39 for males and 40 for females. In this study, the baseline PV status was established as the independent variable. The calculation of PV status was performed retrospectively, and the attending clinicians were blinded to the values during the observation period.

### 2.5. ASV Therapy

In our study, an advanced bi-level positive airway pressure unit, the ASV system, was employed in conjunction with a custom-fitted full-face mask. This sophisticated device autonomously analyzes the patient’s breathing pattern and offers synchronized airway pressure support through the implementation of logical algorithms [8].

The ASV system was configured to provide a foundational positive end-expiratory pressure of 5 cm H_2_O. Additionally, it was programmed to deliver appropriate minimum and maximum inspiratory support, staying within the manufacturer’s stipulated range of 3 to 10 cm H_2_O. The expiratory positive airway pressure was adjustable, ranging from 1 to 5 cm H_2_O, with due consideration to the patient’s comfort.

ASV therapy was administered for a duration exceeding four hours each night, and its continuation was contingent on the amelioration of patients’ congestion or other relevant clinical considerations.

### 2.6. Mid-Term Clinical Data

Demographic details, comorbidities, laboratory findings, medication profiles, and echocardiographic data collected immediately before the commencement of ASV therapy were obtained and constitute the baseline characteristics of the study cohort. Primary emphasis was placed on obtaining plasma BNP levels at baseline and during the three-month follow-up. Additionally, several clinical parameters were monitored and assessed at the three-month mark.

### 2.7. Statistical Analyses

Continuous variables were presented as medians with interquartile ranges (25th percentile to 75th percentile), whereas categorical variables were represented using numbers and corresponding percentages. Patients were stratified into two categories based on the median value of the calculated baseline PV status: a high-PV group and a low-PV group. Comparisons between the two groups for baseline continuous variables were conducted using the Mann–Whitney U test, and for baseline categorical variables, the Chi-square test or Fischer’s exact test was applied.

The independent variable in this analysis was defined as the baseline PV status. The primary objective was to ascertain a decrease in the logarithm of plasma BNP exceeding 0.10 during the three-month ASV therapy. The trajectory of plasma BNP levels over three months was assessed separately for each group (i.e., the high-PV group and the low-PV group) through the Wilcoxon signed-rank test.

To evaluate the impact of the baseline PV status on the primary outcome, a logistic regression analysis was carried out. The analysis was adjusted for potential confounding variables with a significance level (*p*) of less than 0.10 in the univariable analysis. Trends in other clinical parameters, such as the eGFR, were similarly evaluated using the Wilcoxon signed-rank test.

All statistical analyses were conducted using SPSS version 23, and statistical significance was considered at *p*-values less than 0.05.

## 3. Results

### 3.1. Baseline Characteristics

A total of 66 patients were included in this retrospective study (Table 1). All patients had congestive heart failure and received ASV therapy for at least three months. Median age was 66 (59, 74) years old and 53 (80%) were male. The median logarithm of plasma BNP was 2.24 (2.05, 2.60) pg/mL and the median left ventricular ejection fraction was 33% (23%, 47%). All patients received guideline-directed medical therapy as tolerated. Median equivalent dose of furosemide was 20 (0, 30) mg/day.

### 3.2. PV Status

**Figure 1:**
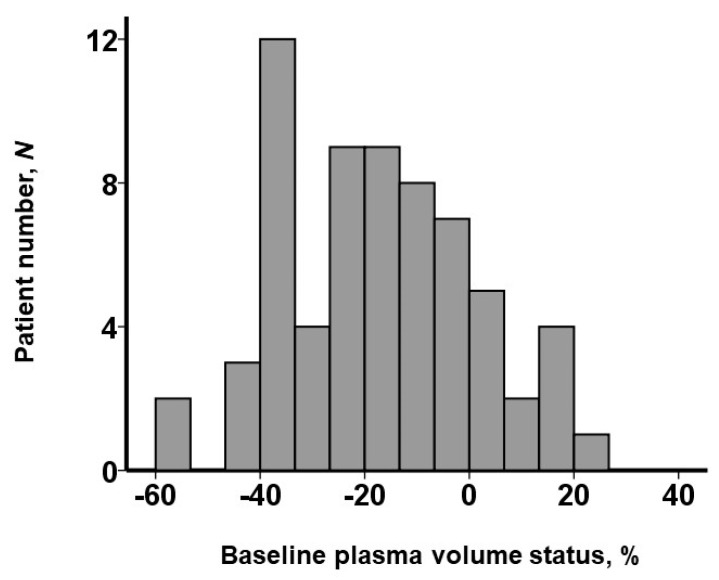
Distribution of baseline plasma volume status.

Baseline PV status was distributed widely with a median value of −16.9% (−29.9%, −4.5%) (Figure 1). Patients were divided into two groups—a high-PV group and a low-PV group—using this median value. Several baseline characteristics were different between the two groups (Table 1). The high-PV group had more advanced anemia, hypoalbuminemia, and more impaired renal function. Plasma BNP levels were not significantly different between the two groups. The prevalence of moderate or greater mitral regurgitation/tricuspid regurgitation was higher in the high-PV group.

### 3.3. The Primary Outcome

**Figure 2:**
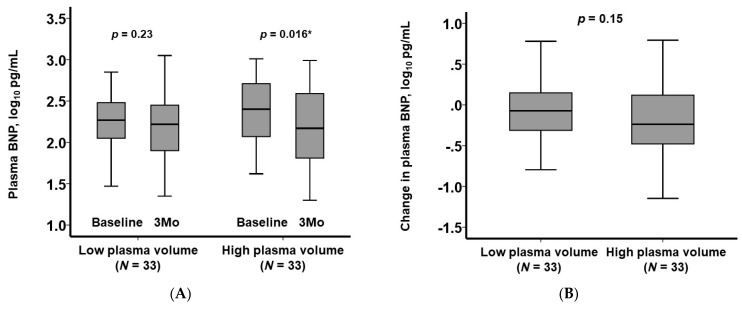
Trend of plasma BNP levels stratified by the plasma volume status (**A**) and a comparison of the change in plasma BNP levels between the high-plasma-volume group and the low-plasma-volume group (**B**). * *p* < 0.05 per Wilcoxon signed-rank test.

The primary focus of our investigation was the alteration in plasma BNP levels throughout the three-month course of ASV therapy. Notably, in the high-PV group, we observed a significant reduction in plasma BNP levels (*p* = 0.016), whereas the low-PV group exhibited no notable change in plasma BNP levels (*p* = 0.23), as depicted in Figure 2A. Although the decline in plasma BNP levels appeared to be more pronounced in the high-PV group compared to the low-PV group, this disparity did not reach statistical significance (*p* = 0.15; Figure 2B).

In examining potential contributing factors, we identified that a lower left ventricular ejection fraction and a higher PV status were both correlated with a noteworthy decrease in plasma BNP levels (*p* < 0.10 for both), as outlined in Table 2. Furthermore, a heightened PV status was independently linked to a substantial reduction in plasma BNP levels, with an adjusted odds ratio of 1.036 (95% confidence interval: 1.01–1.07) (*p* = 0.032).

### 3.4. Trends in Other Clinical Parameters

**Figure 3:**
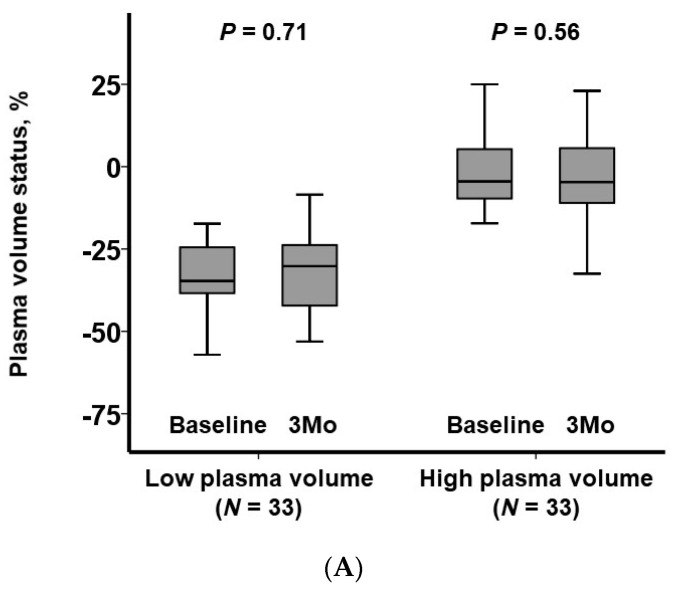

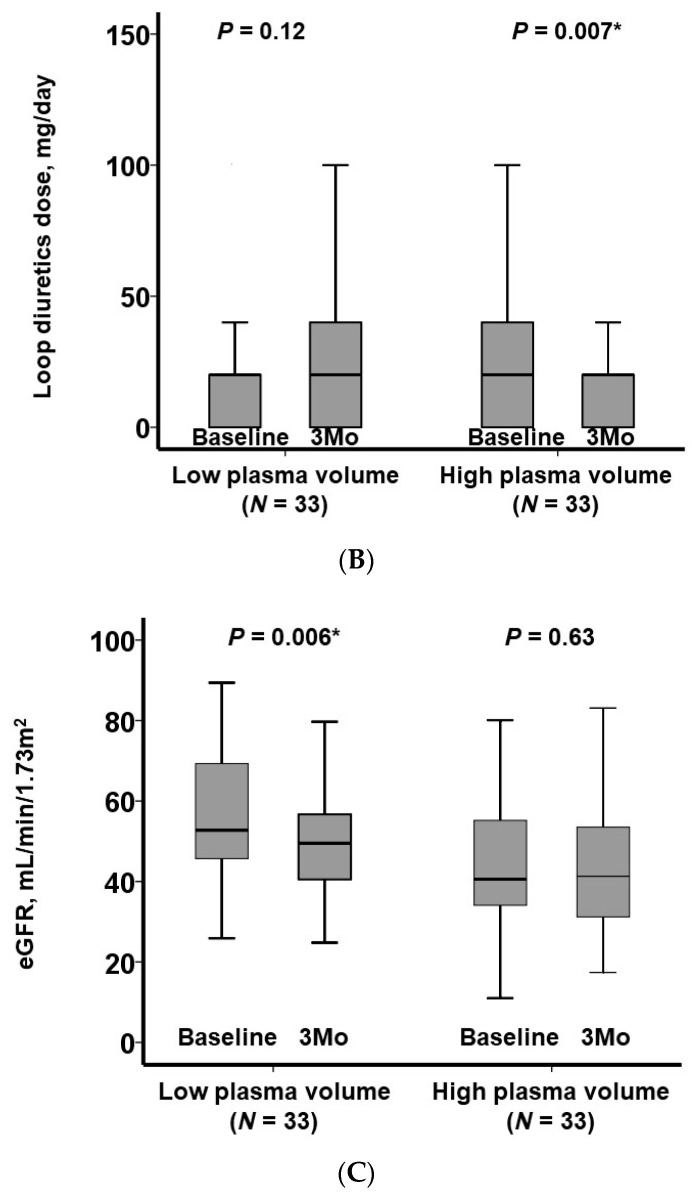
Trends of clinical parameters stratified by plasma volume status: plasma volume status (**A**), the dose of loop diuretics (**B**), and eGFR (**C**). eGFR, estimated glomerular filtration rate. * *p* < 0.05 per Wilcoxon signed-rank test.

PV status exhibited no substantial alterations regardless of the baseline levels (*p* > 0.05 for both groups; Figure 3A). Notably, the high-PV group consistently maintained a higher PV status in comparison to the low-PV group.

The dosage of loop diuretics experienced a reduction in the high-PV group (*p* = 0.007), whereas it remained unaltered in the low-PV group (*p* = 0.12; Figure 3B). In terms of the eGFR, it was preserved in the high-PV group (*p* = 0.63). In contrast, a significant decline in eGFR was observed in the low-PV group (*p* = 0.006; Figure 3C).

## 4. Discussion

Our study aimed to explore the influence of baseline systemic congestion, assessed via the calculated PV status, on the reduction in plasma BNP levels during a three-month course of ASV therapy among patients with congestive heart failure.

The baseline PV status exhibited considerable variability among our patient cohort. We categorized the patients into two groups based on the median value of their baseline PV status. The high-PV group displayed a higher median age, more pronounced renal impairment, and a heightened prevalence of significant valvular diseases in comparison to the low-PV group. Plasma BNP levels exhibited a substantial reduction in the high-PV group but remained unchanged in the low-PV group. Patients in the high-PV group experienced a reduction in the dose of loop diuretics, whereas the dose remained unchanged in the low-PV group. Lastly, eGFR remained preserved in the high-PV group but exhibited a significant decline in the low-PV group.

### 4.1. The Presence of Systemic Congestion

Given the contentious nature of findings surrounding ASV therapy, recent research endeavors have been directed toward identifying the optimal criteria for selecting patients who are likely to benefit from this therapy. Given the significant hemodynamic implications of ASV, including the reduction in preload, enhancement in cardiac unloading, and elevation in cardiac output [12,13,24], investigators have hypothesized that the presence of systemic congestion, a primary target of ASV, is a pivotal determinant of its success.

Prior studies have demonstrated the acute effects of ASV in augmenting cardiac output, particularly in patients exhibiting higher degrees of congestion [15,16,17]. These effects have been assessed through hemodynamic parameters such as right ventricular end-diastolic pressure and pulmonary artery wedge pressure: Hemodynamic congestion was associated with a greater increase in cardiac output during short-term ASV therapy. However, these parameters are reliant on invasive right-heart catheterization, limiting their practicality. Not all candidates of ASV therapy can undergo such an invasive procedure beforehand. Additionally, it remains uncertain whether these acute effects are associated with favorable long-term clinical outcomes.

In response to these challenges, we employed a method to estimate PV status utilizing straightforward clinical parameters and evaluated its impact on mid-term ASV therapy. We did not prefer plasma BNP levels to assess the presence of systemic congestion given their weak correlation. Instead, plasma BNP levels were used as a surrogate marker to quantify the effect of ASV therapy, offering a more accessible and non-invasive means of assessing its impact.

### 4.2. Impact of ASV on Reducing Plasma BNP Levels

The presence of systemic congestion emerged as an independent factor associated with a substantial reduction in plasma BNP levels following three-month ASV therapy. In patients with low-PV status, plasma BNP levels remained unchanged. This observation aligns with the expected mechanism, as ASV therapy diminishes preload and concurrently enhances cardiac output. Consequently, the presence of an adequate preload appears to be a prerequisite for experiencing the benefits of ASV. Administering ASV support to patients without systemic congestion might lead to a reduction in cardiac output, potentially causing hemodynamic deterioration and elevating the risk of cardiovascular complications, as was noted in the SERVE-HF trial [14]. In our study, we set a reduction in the logarithm of plasma BNP exceeding 0.10 as the primary endpoint, as subtle enhancements in heart failure might be attainable through background medications alone.

An intriguing observation was that the high-PV group experienced a reduction in the dosage of loop diuretics and preserved renal function. This finding can be elucidated by considering the intricate cardio-renal relationship. An augmentation in cardiac output could lead to an amelioration in renal blood flow, and the mitigation of systemic congestion may also alleviate renal congestion. The reduction in loop diuretic dosage was likely a consequence of the improvement in systemic congestion and could potentially confer a renoprotective effect.

PV status remained unchanged during mid-term ASV support even in patients with baseline elevated PV status. ASV itself reduces preload by increasing intra-thoracic pressure but may not necessarily reduce the total PV. Further studies are warranted to assess the impact of ASV therapy on the PV status by measuring the trajectory of PV status during ASV support.

### 4.3. Clinical Implication of Our Findings

In clinical guidelines, the identification of systemic congestion is strongly advised as a crucial consideration when contemplating ASV therapy [25]. However, the precise assessment of congestion can be a challenging task. Our study revealed a broad distribution of PV status despite the diligent efforts of attending physicians to evaluate congestion using conventional methods. Instead, the computation of PV status emerged as a valuable tool for gauging the extent of systemic congestion, as demonstrated in our research. Another promising modality is remote dielectric sensing, which offers a non-invasive, rapid assessment of lung fluid levels within a minute [26]. A drawback of this method is its high cost, and it is not widely available at many healthcare institutions. PV status can be calculated easily by several clinical parameters without any equipment or expert technique.

The guidelines suggest that ASV therapy should be discontinued once congestion has been adequately managed [1]. In our study, the high-PV group continued to exhibit elevated PV status. This is the rationale for why we continued ASV therapy in this cohort to treat residual congestion (we emphasize that PV status was blinded to the attending clinicians during ASV therapy). Hence, it is advisable to periodically reevaluate PV status to determine the optimal timing for discontinuing ASV therapy. The normal range of PV status has not yet been established, and further studies are warranted to define optimal cutoff to terminate ASV therapy.

The optimal threshold for PV status to identify good candidates of ASV therapy remains uncertain. Although we employed the median value to divide the patient cohort, it is conceivable that other suitable cutoffs may be identified in future research. In our study, we used plasma BNP levels as a surrogate marker for clinical outcomes. The next area of concern would be to explore the prognostic impact of PV status on hard endpoints such as mortality, heart failure readmission, or any other symptom-related parameters.

### 4.4. Strength of This Study

The presence of systemic congestion, a primary target of ASV therapy, is pivotal for its efficacy. Administering ASV therapy to individuals without systemic congestion may inappropriately diminish preload, leading to a decrease in cardiac output and potentially exacerbating clinical outcomes. However, accurately assessing the presence of systemic congestion poses a clinical challenge.

We demonstrated for the first time that systemic congestion, quantified by PV status, was associated with a significant reduction in plasma BNP levels—a surrogate marker for heart failure improvement—during mid-term ASV therapy. Based on our findings, we strongly recommend that clinicians calculate PV status before considering the applicability of ASV therapy for potential candidates with congestive heart failure.

### 4.5. Study Limitations

This investigation is limited by its single-center retrospective design, focusing on a moderate-sized cohort. Some statistical findings might have attained significance with a larger sample size. Our study exclusively included patients who sustained ASV therapy for more than three months, omitting those who discontinued ASV within the initial three months. Although we adhered to the principle of maintaining stable medication dosages, except for loop diuretics, the potential influence of these medications should not be underestimated. Although the administration of anti-heart failure agents did not exhibit a statistically significant association with the primary outcome, the impact of these medications remains a relevant consideration. On the contrary, it is ethically challenging not to administer ASV therapy in patients with congestive heart failure refractory to medications for the establishment of the control group. Similarly, ASV therapy without any heart failure medications is a form of off-label use and hardly recommended.

The study’s duration spanned only three months, and questions regarding the influence of PV status on long-term ASV therapy effects constitute a future research concern. On the contrary, ASV therapy should be terminated when congestion is ameliorated and such long-term ASV therapy may not be realistic.

Several clinical parameters were lacking that could have strengthened our findings, such as the presence of proteinuria. Successful ASV therapy has potential to improve renal function due to incremental renal flow. Proteinuria may have improved during ASV therapy in patients with baseline elevated PV status.

## 5. Conclusions

The presence of systemic congestion through the assessment of PV status was linked to a noteworthy reduction in plasma BNP levels throughout three-month ASV therapy. Future research endeavors should focus on determining the optimal threshold for PV status and evaluating its long-term prognostic implications, especially concerning hard endpoints.

## Figures and Tables

**Table 1:** Baseline characteristics.

	Total (*N* = 66)	High Plasma Volume (*N* = 33)	Low Plasma Volume (*N* = 33)	*p*-Value
Demographics				
Age, years	66 (59, 74)	73 (65, 78)	61 (54, 68)	<0.001 *
Male sex	53 (80%)	23 (70%)	30 (91%)	0.030 *
Body mass index, kg/m^2^	23.1 (20.0, 26.8)	20.3 (18.8, 22.6)	26.8 (24.0, 29.3)	<0.001 *
Comorbidity				
Hypertension	26 (39%)	14 (42%)	12 (36%)	0.61
Diabetes mellitus	22 (33%)	6 (18%)	16 (48%)	0.009 *
Atrial fibrillation	30 (45%)	14 (42%)	16 (48%)	0.40
Coronary artery disease	25 (38%)	14 (42%)	11 (33%)	0.31
History of stroke	5 (8%)	3 (9%)	2 (6%)	0.64
Peripheral artery disease	11 (17%)	6 (18%)	5 (15%)	0.74
Laboratory data				
Hemoglobin, g/dL	12.9 (11.4, 14.1)	11.5 (10.3, 12.4)	14.0 (13.2, 15.4)	<0.001 *
Hematocrit, %	38.7 (35.2, 42.7)	35.3 (31.1, 37.9)	42.4 (40.6, 45.9)	<0.001 *
Serum albumin, g/dL	3.9 (3.6, 4.1)	3.7 (3.4, 3.9)	4.1 (3.9, 4.3)	<0.001 *
Serum sodium, mEq/L	139 (137, 141)	138 (135, 141)	140 (138, 141)	0.065
Serum potassium, mEq/L	4.3 (4.0, 4.5)	4.4 (4.2, 4.7)	4.2 (4.0, 4.4)	0.053
Serum total bilirubin, mg/dL	0.7 (0.5, 0.9)	0.6 (0.5, 0.8)	0.8 (0.6, 1.0)	0.039 *
eGFR, mL/min/1.73 m^2^	48.4 (38.5, 59.0)	41.1 (33.8, 55.3)	50.9 (45.7, 66.6)	0.003 *
Plasma BNP, log_10_ pg/mL	2.24 (2.05, 2.60)	2.37 (2.06, 2.71)	2.21 (1.98, 2.53)	0.26
Echocardiography				
LVDd, mm	62 (55, 69)	58 (52, 69)	65 (60, 74)	0.062
LVEF, %	33 (23, 47)	34 (24, 54)	32 (23, 39)	0.49
Left atrial diameter, mm	47 (41, 53)	43 (40, 51)	49 (43, 53)	0.028 *
Moderate or greater MR	20 (30%)	15 (45%)	5 (15%)	0.011 *
Moderate or greater TR	16 (24%)	13 (39%)	3 (9%)	0.006 *
Medication				
Beta-blocker	52(79%)	25 (76%)	27 (82%)	0.38
RAS inhibitor	58 (88%)	28 (85%)	30 (91%)	0.45
MRA	38 (58%)	16 (48%)	22 (67%)	0.11
SGLT2 inhibitor	26 (39%)	12 (36%)	14 (42%)	0.61
Dose of loop diuretics, mg/day	20 (0, 30)	20 (0. 40)	20 (0, 20)	0.24
Plasma volume status, %	−16.9 (−29.9, −4.5)	−4.7 (−10.8, 3.4)	−34.7 (−38.3, −23.6)	<0.001 *

Continuous variables were presented as the median (25% interquartile, 75% interquartile) and compared between the two groups using the Mann–Whitney U test. Categorical variables were presented as numbers and percentages and compared between the two groups using the Chi-square test or Fischer’s exact test. eGFR, estimated glomerular filtration rate; BNP, B-type natriuretic peptide; LVDd, left ventricular end-diastolic diameter; LVEF, left ventricular ejection fraction; MR, mitral regurgitation; TR, tricuspid regurgitation; RAS, renin-angiotensin system; MRA, mineralocorticoid receptor antagonist. * *p* < 0.05.

**Table 2:** Potential factors associated with significant reduction in plasma BNP levels during 3-month ASV therapy.

	Univariable Analysis		Multivariable Analysis	
	Odds Ratio (95% CI)	*p*-Value	Odds Ratio (95% CI)	*p*-Value
Age, years	1.02 (0.98–1.06)	0.43		
Atrial fibrillation	0.58 (0.22–1.56)	0.28		
Coronary artery disease	0.59 (0.21–1.64)	0.31		
eGFR, mL/min/1.73 m^2^	0.98 (0.95–1.01)	0.12		
Plasma BNP, log_10_ pg/mL	2.53 (0.75–8.55)	0.14		
LVDd, mm	1.02 (0.98–1.07)	0.34		
LVEF, %	0.97 (0.94–1.01)	0.098	0.97 (0.94–1.01)	0.058
Moderate or greater MR	1.33 (0.46–3.88)	0.6		
Moderate or greater TR	1.30 (0.42–4.07)	0.65		
Beta-blocker	2.32 (0.64–8.33)	0.2		
RAS inhibitor	1.35 (0.30–6.20)	0.7		
MRA	1.08 (0.40–2.89)	0.88		
SGLT2 inhibitor	1.06 (0.42–2.67)	0.67		
Dose of loop diuretics, mg/day	1.01 (0.99–1.02)	0.33		
Plasma volume status, %	1.03 (1.01–1.06)	0.046 *	1.036 (1.01–1.07)	0.032 *

CI, confidence interval; eGFR, estimated glomerular filtration rate; BNP, B-type natriuretic peptide; LVDd, left ventricular end-diastolic diameter; LVEF, left ventricular ejection fraction; MR, mitral regurgitation; TR, tricuspid regurgitation; RAS, renin-angiotensin system; MRA, mineralocorticoid receptor antagonist. * *p* < 0.05 per logistic regression analysis. Variables with *p* < 0.10 were included in the multivariable analysis.

## Data Availability

Data are available from the corresponding author upon reasonable reason.

## References

[1] Tsutsui H., Ide T., Ito H., Kihara Y., Kinugawa K., Kinugawa S., Makaya M., Murohara T., Node K., Saito Y. (2021). JCS/JHFS 2021 Guideline Focused Update on Diagnosis and Treatment of Acute and Chronic Heart Failure. Circ. J..

[2] Heidenreich P.A., Bozkurt B., Aguilar D., Allen L.A., Byun J.J., Colvin M.M., Deswal A., Drazner M.H., Dunlay S.M., Evers L.R. (2022). 2022 AHA/ACC/HFSA Guideline for the Management of Heart Failure: A Report of the American College of Cardiology/American Heart Association Joint Committee on Clinical Practice Guidelines. Circulation.

[3] McDonagh T.A., Metra M., Adamo M., Gardner R.S., Baumbach A., Bohm M., Burri H., Butler J., Celutkiene J., Chioncel O. (2021). 2021 ESC Guidelines for the diagnosis and treatment of acute and chronic heart failure. Eur. Heart J..

[4] Chen J., Aronowitz P. (2022). Congestive Heart Failure. Med. Clin. N. Am..

[5] Patel A., Perez I., Rabiei-Samani S. (2021). What Is Adaptive Servo-Ventilation (ASV)?. Am. J. Respir. Crit. Care Med..

[6] Imamura T., Narang N., Kinugawa K. (2022). Adaptive Servo-Ventilation as a Novel Therapeutic Strategy for Chronic Heart Failure. J. Clin. Med..

[7] Koyama T., Watanabe H., Kobukai Y., Makabe S., Munehisa Y., Iino K., Kosaka T., Ito H. (2010). Beneficial effects of adaptive servo ventilation in patients with chronic heart failure. Circ. J..

[8] Kasai T., Usui Y., Yoshioka T., Yanagisawa N., Takata Y., Narui K., Yamaguchi T., Yamashina A., Momomura S.I. (2010). Effect of flow-triggered adaptive servo-ventilation compared with continuous positive airway pressure in patients with chronic heart failure with coexisting obstructive sleep apnea and Cheyne-Stokes respiration. Circ. Heart Fail..

[9] Haruki N., Takeuchi M., Kaku K., Yoshitani H., Kuwaki H., Tamura M., Abe H., Okazaki M., Tsutsumi A., Otsuji Y. (2011). Comparison of acute and chronic impact of adaptive servo-ventilation on left chamber geometry and function in patients with chronic heart failure. Eur. J. Heart Fail..

[10] Yoshihisa A., Suzuki S., Miyata M., Yamaki T., Sugimoto K., Kunii H., Nakazato K., Suzuki H., Saitoh S., Takeishi Y. (2012). “A single night” beneficial effects of adaptive servo-ventilation on cardiac overload, sympathetic nervous activity, and myocardial damage in patients with chronic heart failure and sleep-disordered breathing. Circ. J..

[11] Kasai T., Kasagi S., Maeno K., Dohi T., Kawana F., Kato M., Naito R., Ishiwata S., Ohno M., Yamaguchi T. (2013). Adaptive servo-ventilation in cardiac function and neurohormonal status in patients with heart failure and central sleep apnea nonresponsive to continuous positive airway pressure. JACC Heart Fail..

[12] Momomura S., Seino Y., Kihara Y., Adachi H., Yasumura Y., Yokoyama H. (2015). Adaptive servo-ventilation therapy using an innovative ventilator for patients with chronic heart failure: A real-world, multicenter, retrospective, observational study (SAVIOR-R). Heart Vessels..

[13] Momomura S., Seino Y., Kihara Y., Adachi H., Yasumura Y., Yokoyama H., Wada H., Ise T., Tanaka K. (2015). Adaptive servo-ventilation therapy for patients with chronic heart failure in a confirmatory, multicenter, randomized, controlled study. Circ. J..

[14] Cowie M.R., Woehrle H., Wegscheider K., Angermann C., d’Ortho M.P., Erdmann E., Levy P., Simonds A.K., Somers V.K., Zannad F. (2015). Adaptive Servo-Ventilation for Central Sleep Apnea in Systolic Heart Failure. N. Engl. J. Med..

[15] Yamada S., Sakakibara M., Yokota T., Kamiya K., Asakawa N., Iwano H., Yamada S., Oba K., Tsutsui H. (2013). Acute hemodynamic effects of adaptive servo-ventilation in patients with heart failure. Circ. J..

[16] Yagi S., Akaike M., Iwase T., Kusunose K., Niki T., Yamaguchi K., Koshiba K., Taketani Y., Tomita N., Yamada H. (2011). Acute hemodynamic effects of adaptive servo ventilation in patients with pulmonary hypertension. Int. J. Cardiol..

[17] Yamada S., Sakakibara M., Matsushima S., Saito A., Homma T., Fukushima A., Masaki Y., Watanabe M., Mitsuyama H., Yokoshiki H. (2011). Successful termination of recurrent ventricular arrhythmias by adaptive servo-ventilation in a patient with heart failure. J. Cardiol. Cases.

[18] Ling H.Z., Flint J., Damgaard M., Bonfils P.K., Cheng A.S., Aggarwal S., Velmurugan S., Mendonca M., Rashid M., Kang S. (2015). Calculated plasma volume status and prognosis in chronic heart failure. Eur. J. Heart Fail..

[19] Kawai T., Nakatani D., Yamada T., Sakata Y., Hikoso S., Mizuno H., Suna S., Kitamura T., Okada K., Dohi T. (2021). Clinical impact of estimated plasma volume status and its additive effect with the GRACE risk score on in-hospital and long-term mortality for acute myocardial infarction. Int. J. Cardiol. Heart Vasc..

[20] Tamaki S., Yamada T., Morita T., Furukawa Y., Iwasaki Y., Kawasaki M., Kikuchi A., Kawai T., Seo M., Abe M. (2019). Prognostic Value of Calculated Plasma Volume Status in Patients Admitted for Acute Decompensated Heart Failure—A Prospective Comparative Study with Other Indices of Plasma Volume. Circ. Rep..

[21] Grodin J.L., Philips S., Mullens W., Nijst P., Martens P., Fang J.C., Drazner M.H., Tang W.H.W., Pandey A. (2019). Prognostic implications of plasma volume status estimates in heart failure with preserved ejection fraction: Insights from TOPCAT. Eur. J. Heart Fail..

[22] Martens P., Nijst P., Dupont M., Mullens W. (2019). The Optimal Plasma Volume Status in Heart Failure in Relation to Clinical Outcome. J. Card. Fail..

[23] McKee P.A., Castelli W.P., McNamara P.M., Kannel W.B. (1971). The natural history of congestive heart failure: The Framingham study. N. Engl. J. Med..

[24] Seino Y., Momomura S., Kihara Y., Adachi H., Yasumura Y., Yokoyama H., SAVIOR-C Investigators (2015). Effects of adaptive servo-ventilation therapy on cardiac function and remodeling in patients with chronic heart failure (SAVIOR-C): Study protocol for a randomized controlled trial. Trials.

[25] Tsutsui H., Isobe M., Ito H., Ito H., Okumura K., Ono M., Kitakaze M., Kinugawa K., Kihara Y., Goto Y. (2019). JCS 2017/JHFS 2017 Guideline on Diagnosis and Treatment of Acute and Chronic Heart Failure—Digest Version. Circ. J..

[26] Imamura T., Narang N., Kinugawa K. (2023). Clinical implications of remote dielectric sensing system to estimate lung fluid levels. J. Cardiol..

